# *CaZingipain2* Acts Positively in Pepper (*Capsicum annuum* L.) Immunity against *R. solanacearum*

**DOI:** 10.3390/plants13182552

**Published:** 2024-09-11

**Authors:** Ruijie Wu, Zhen Wu, Yalin Qing, Chenfeng Duan, Yiling Guo, Xujing Zhang, Ronghua Huang, Shuilin He, Ailian Qiu

**Affiliations:** 1 College of Agriculture, Fujian Agriculture and Forestry University, Fuzhou 350002, China; 2190102009@fafu.edu.cn (R.W.); baiwenbuyanzhe@163.com (Z.W.); qingyalin2023@163.com (Y.Q.); 18558768917@163.com (Y.G.); 000q010003@fafu.edu.cn (R.H.); 2 College of Life Science, Fujian Agriculture and Forestry University, Fuzhou 350002, China; dcf3126181949@163.com (C.D.); zxjgzyx0709@163.com (X.Z.)

**Keywords:** *R. solanacearum*, pepper, ginger protease, Zingipain

## Abstract

Bacterial wilt caused by *Ralstonia solanacearum* is one of the most important diseases in solanaceous plants, including peppers. It generally tends to be more serious under warm−temperature and moist (WM) conditions than at moist room−temperature (RM) conditions. Although immunity mechanisms at room temperature have been intensively studied, the mechanisms underlying WM conditions remain poorly understood. Herein, the pepper cysteine protease CaZingipain2 was expressed and functionally characterized in pepper immunity against *R. solanacearum* at WM conditions and at room temperature. The results showed that CaZingipain2 localized to the nucleus and was upregulated at the transcript level in pepper plants upon *R. solanacearum* infection under WM conditions (RSWM). Virus−induced gene silencing of *CaZingipain2* significantly increased the susceptibility of pepper plants to RSWM, and was coupled with the downregulation of *CaPRP1* and *CaMgst3*, which are specifically related to pepper immunity against RSWM, according to our previous studies, while its overexpression significantly reduced the susceptibility of *N. benethamiana* plants to RSWM compared to that of wild−type plants. In addition, our data showed that CaZingipain2 also acts positively in pepper immunity against *R. solanacearum* infection at room temperature by upregulating the SA− and JA−responsive PR genes, including *CaNPR1* and *CaDEF1*. All these results indicate that *CaZingipain2* improves pepper immunity against *R. solanacearum* under WM conditions and at room temperature by regulating different PR genes.

## 1. Introduction

Plants are often attacked by pathogens during their growth. Under the pressure of natural selection, plants have evolved a sophisticated immune system comprising two interconnected branches, known as pathogen−associated molecular pattern (PAMP) −triggered immunity (PTI) and effector−triggered immunity (ETI) [1,2,3]. Many studies have revealed crosstalk and cooperation between ETI and PTI [4]. They share common signaling components, such as kinases, transcription factors, transcriptional regulators, and other proteins, that regulate various biological processes potentially related to plant immunity [5,6]. The determination and functional characterization of these proteins might help in elucidating the mechanisms underlying plant immunity. Cysteine proteases have been widely found to be involved in plant immunity as a central hub common to both PTI and ETI [7,8,9,10] and, thus, they are targeted by effectors from pathogens to suppress plants’ innate immunity [9,11,12,13]. Cysteine proteases have a variety of roles in PTI. When plants sense PAMPs, they quickly express cysteine proteases to activate defense gene expression and cell wall reinforcement. For example, in *Zea mays*, an immune signaling peptide released from a propeptide via PLCP activity activates salicylic acid signaling [14]. In *Arabidopsis thaliana*, the cysteine protease Caspase−1−like protein limits the spread of pathogens by regulating programmed cell death and defense signaling [15]. ETI generally exhibits a stronger and more persistent immune response than PTI, which includes oxidative bursts, programmed cell death, and systemic acquired resistance (SAR). Cysteine proteases also play a key role in ETI. By regulating cell death mechanisms, such as the hypersensitive response (HR), they ensure that pathogens are contained early in infection [16].

Cysteine proteases also have important functions at multiple stages of plant development. They are involved in seed germination, leaf senescence, and flower organ shedding [17]. During seed germination, cysteine proteases provide amino acids and energy to embryos by degrading storage proteins. In leaf senescence, these enzymes maintain intracellular homeostasis by regulating protein degradation, thereby promoting the orderly aging of leaves. Cysteine proteases are also involved in plant hormone signaling pathways. Studies have shown that the cleavage of Ca^2+^ transporters by cysteine proteases can alter Ca^2+^ signaling [18]. Overall, cysteine proteases are found in a variety of organisms and are involved in protein degradation and conversion, programmed cell death, and immunity [19,20]. Zingipain was first extracted and purified from ginger, so it was named ginger protease. However, research on Zingipain has mainly focused on major human diseases and the food industry [21,22], and has rarely been reported in plants.

Plant–pathogen interactions might be affected by environmental conditions [23]. Ambient temperature has a highly significant influence on the plant immune system [24]. Warm−temperature and moist (WM) conditions are amongst the most frequently occurring environmental conditions during the growth and development of solanaceous plants, including the pepper, tomato, and tobacco, in tropical and subtropical regions, possibly promoting the growth and development of pathogens and repressing plant immunity, thereby causing serious diseases in these crops [25,26]. Serious diseases under WM conditions might have exerted selective pressure on plants and profoundly affected the immunity that has evolved in tropical and subtropical regions where WM conditions frequently occur. Other studies have also found that plants develop some unique resistance to disease at higher temperatures, including against fungi, nematodes, and viruses [27,28,29]. Bacterial wilt is a common soil−borne disease that is destructive to the production of solanaceous plants [30]. Bacterial wilt is caused by the *R. solanacearum* species complex (RSSC), which causes disease by blocking the xylem [31,32]. The RSSC can also damage a host’s immune system and cell activity by secreting effector factors and toxic substances [31]. Plant immunity against *R. solanacearum* under WM conditions differs significantly from that at room temperature, even under moist conditions (RM) [33]. However, plant immunity under WM conditions remains poorly understood compared to that at room temperature; in particular, how plants coordinate immunity against a given pathogen under different environments remains unexplored.

In the present study, we report that CaZingipain2 transcriptionally responds to *Ralstonia solanacearum* infection under WM conditions and acts as a positive regulator of pepper immunity against *R. solanacearum* infection under WM conditions.

## 2. Results

### 2.1. CaZingipain2 Upregulation in Pepper Plants upon R. Solanacearum Inoculation under WM Conditions

**Figure 1:**
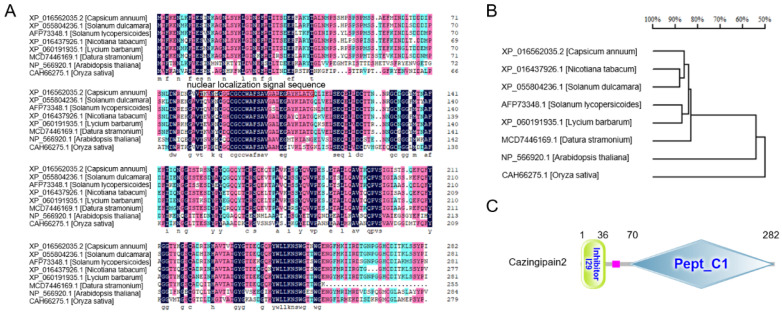
Sequence of CaZingipain2 relative to its orthologs in other plant species. (**A**) Multiple sequence alignment of CaZingipain2 with its orthologs from other plant species, including *Solanum dulcamara*, *Solanum lycopersicoides*, *Nicotiana tabacum*, *Lycium barbarum*, *Datura stramonium*, *Arabidopsis thaliana*, and *Oryza sativa* (performed using DNAMAN8). Blue shaded parts, 50−75%, red shaded parts, 75−100%, black shaded parts, 100%. (**B**) Phylogenetic analysis via MEGA11 (mega software) revealed that CaZingipain2 shares more than 80% sequence identity with Zingipain2−like in *Solanum dulcamara.* (**C**) By domain searching, Pept C1 domain was found in amino acid sequence of CaZingipain2 by SMART 6 (http://smart.embl.de/) (accessed on 1 March 2019).

CaZingipain2, a gene encoding Zingipain in pepper that is upregulated upon *R. solanacearum* inoculation under WM conditions, caught our attention in an RNA−seq dataset of pepper plants upon *R. solanacearum* inoculation under WM conditions [33]. A sequence analysis showed that it has an ORF encoding a 283−amino−acid protein, it belongs to the papain family (Figure 1C), and its sequence has similarity to Zingipain2−like (*Solanum dulcamara*, XP_055804236.1), cysteine protease (*Solanum lycopersicoides*, AFP73348.1), tobacco Zingipain2−like (*Nicotiana tabacum*, XP_016437926.1), Zingipain2−like (*Lycium barbarum*, XP_060191935.1), Zingipain2−like (*Datura stramonium*, MCD7446169.1), the *Arabidopsis thaliana* cysteine proteinase superfamily protein (*Arabidopsis thaliana*, NP_566920.1), and rice (*Oryza sativa*, CAH66275.1). These proteins were compared based on their amino acid homology (Figure 1A), and an evolutionary tree was constructed (Figure 1B). Among its orthologs in different plant species, CaZingipain2 exhibited the highest sequence similarity to Zingipain2−like in *Solanum dulcamara*.

### 2.2. CaZingipain2 Localized to Nuclei in Epidermal Cells of Nicotiana benthamiana Leaves

**Figure 2:**
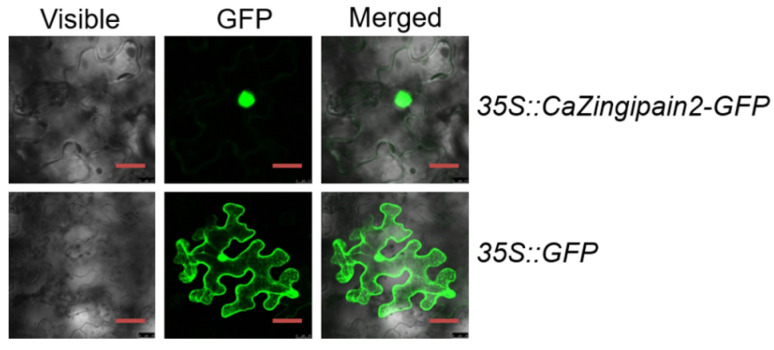
CaZingipain2 is localized in the nucleus. After transient overexpression of CaZingipain2 in *Nicotiana benthamiana* epidermal cells by agroinfiltration, CaZingipain2 was shown to target the nucleus. Bars = 50 μm.

According to the prediction of the cNLS Mapper website (https://nls-mapper.iab.keio.ac.jp/cgi-bin/NLS_Mapper_form.cgi), (accessed on 1 March 2019) the amino acid sequence of CaZingipain2 has a nuclear localization signal sequence (TQVKHQGQCGCCWAFSAVGALEGAYKLATG) (position marked by the red box in Figure 1A). It appeared to be a nuclear protein so, to test this possibility, we generated a CaZingipain2–GFP fusion driven by the constitutive *CaMV35S* promoter (*p35S::CaZingipain2*−*GFP*) using *p35S::GFP* as the negative control. By agroinfiltration, the subcellular location of CaZingipain2−GFP was detected in the epidermal cells of *N. benthamiana* leaves. The results showed that CaZingipain2 localizes exclusively in the nuclei, while GFP occurs in multiple subcellular compartments, including the cytoplasm and nuclei. This suggests that CaZingipain2 is nuclear localized (Figure 2).

### 2.3. CaZingipain2 Is Upregulated upon R. solanacearum Infection under RM and WM Conditions

**Figure 3:**
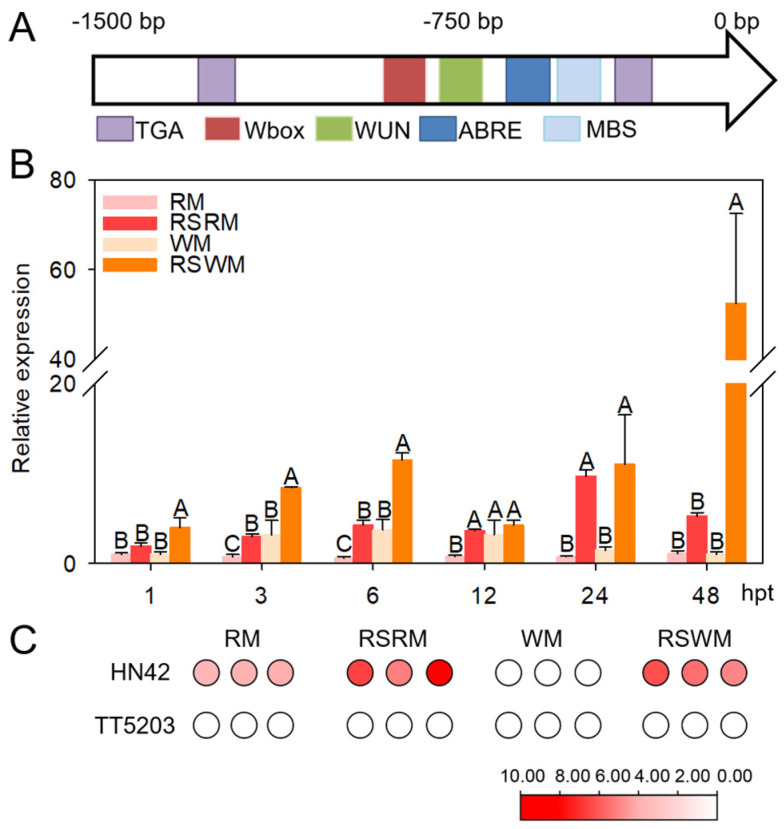
Expression of *CaZingipain2*. (**A**) Distribution of cis elements in *CaZingipain2* promoter. (**B**) Transcript levels of *CaZingipain2* in leaves of pepper plants challenged with RSI. Transcript level of each tested gene was normalized to *CaActin*. Different letters indicate significant differences (*p* < 0.01) as determined using Fisher’s protected LSD test. (**C**) Transcript levels of *CaZingipain2* in roots of two inbred pepper lines, HN42 and TT5203, under RSI at RM and WM. Relative expression levels were normalized based on FPKM (fragments per kilobase of exon model per million mapped fragments) generated by RNA−seq. Note that red indicates strongest expression level.

An RNA−seq originally showed that CaZingipain2 is upregulated in pepper plants upon *R. solanacearum* inoculation under WM conditions (37 °C, more than 50% soil water content and 80% air humidity). To test whether this was the case, we first checked the cis elements in the 1500 bp promoter region using PlantCARE online. Stress− or pathogen−responsive cis elements, such as TGA, W−box, the ABA−responsive element, and the WUN element, were found (Figure 3A), implying the possible involvement of CaZingipain2 in pepper plants’ response to pathogen infection or abiotic stresses. We further tested *CaZingipain2* expression in pepper plants upon *R. solanacearum* inoculation under different conditions by qRT−PCR, and found that it was upregulated not only under RM conditions (28 °C, more than 50% soil water content and 80% air humidity), but also under WM conditions compared to the mock treatment (Figure 3B). Furthermore, by checking the RNA−seq data from two inbred pepper lines with different levels of disease resistance, it was found that *CaZingipain2* exhibited a higher transcript level of HN42, the inbred line with a high level of disease resistance under WM conditions, but *CaZingipain2* was not expressed in the disease−susceptible inbred pepper line TT5203 (Figure 3C).

### 2.4. Silencing of CaZingipain2 Enhances Susceptibility of Pepper to R. solanacearum

**Figure 4:**
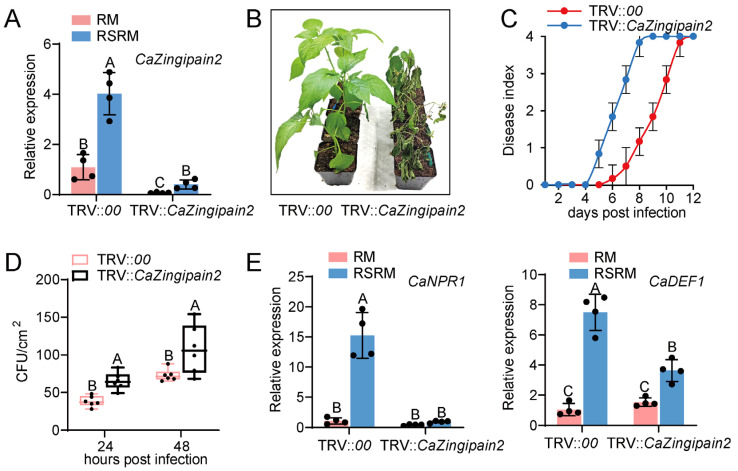
*CaZingipain2* silencing significantly reduced pepper immunity against *R. solanacearum* at RM. (**A**) Success of *CaZingipain2* silencing in pepper plants (TRV::*CaZingipain2*) by qRT−PCR using specific primer pair for *CaZingipain2*. (**B**) Compared to control plants (TRV::*00)*, TRV::*CaZingipain2* exhibited increased susceptibility to RSRM. (**C**) TRV::*CaZingipain2* pepper plants exhibited greater dynamic disease index from 5 to 12 dpi than TRV::*00* plants at RM. Ten pepper plants were subjected to analysis of disease indices over time (*n* = 10), which indicated a significant difference between TRV::*CaZingipain2* and TRV::*00*, with *p* < 0.001. (**D**) The TRV::*CaZingipain2* plants promoted *R. solanacearum* growth at 24 and 48 hpi at RM. (**E**) TRV::*CaZingipain2* plants exhibited reduced expression of SA− and JA−responsive immunity genes, including *CaNPR1* and *CaDEF1*. In (**A**,**D**,**E**), the transcriptional level of each tested gene is normalized to that of *CaActin*. Dots represent biological replicates. Different letters indicate significant differences (*p* < 0.01) as determined using Fisher’s protected LSD test.

To assess the role of pepper CaZingipain2’s response to *R. solanacearum*, we used the virus−induced gene silencing (VIGS) technique to generate *CaZingipain2*−silenced pepper seedlings. Compared to the TRV::*00* plants, the transcriptional levels of Cazingipain2 were greatly reduced in the *CaZingipain2*−silenced plants (Figure 4A), indicating the success of *CaZingipain2* silencing. We then inoculated TRV::*00* and TRV::*CaZingipain2* plants with *R. solanacearum* under RM conditions, and observed that the *CaZingipain2*−silenced plants showed a more pronounced wilted phenotype than the control plants under the two tested conditions (Figure 4B). Consistent with this result, the *CaZingipain2*−silenced plants exhibited a higher disease index from 5 to 12 days post−infection (dpi) (Figure 4C), and higher levels of *R. solanacearum* growth and accumulation than the control plants at 24 and 48 hpi (Figure 4D).

Under RM conditions, the positive role of *CaZingipain2* in pepper immunity against *R. solanacearum* infection was related to SA−responsive *CaNPR1* and JA−responsive *CaDEF1*, since the upregulation of these two genes by *R. solanacearum* infection under RM conditions was significantly reduced by *CaZingipain2* silencing (Figure 4E).

**Figure 5:**
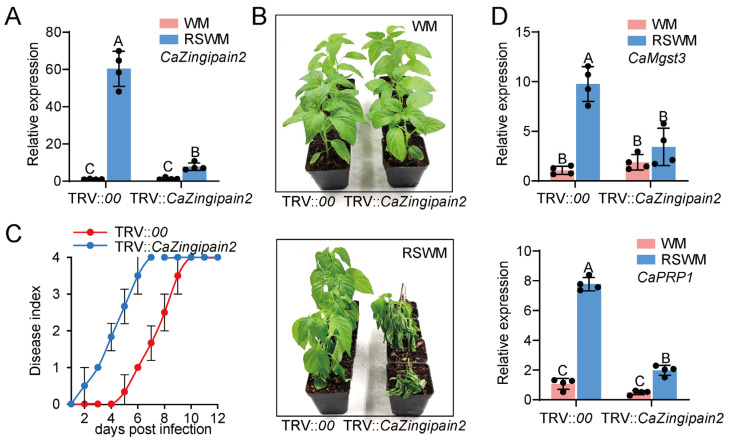
*CaZingipain2* silencing significantly reduced pepper immunity against *R. solanacearum* at WM. (**A**) Success of *CaZingipain2* silencing in pepper plants (TRV::*Cazingipain2*) by qRT−PCR using specific primer pair for *CaZingipain2*. (**B**) Compared to TRV::*00*, TRV::*Cazingipain2* exhibited higher susceptibility to RSWM, but TRV::*CaZingipain2* and control plants did not exhibit altered phenotypic damage at WM. (**C**) TRV::*CaZingipain2* pepper plants exhibited greater dynamic disease index from 2 to 12 dpi than TRV::*00* plants at WM. Ten pepper plants were subjected to analysis of disease indices over time (*n* = 10), which indicated a significant difference between TRV::*CaZingipain2* and TRV::*00* with *p* < 0.001. (**D**) TRV::*CaZingipain2* plants exhibited reduced expression of warm−temperature−responsive immunity genes, including *CaMgst3* and *CaPRP1*. In (**A**,**D**), transcriptional level of each tested gene is normalized to that of *CaActin*. Dots represent four biological replicates (*n* = 4). Different letters indicate significant differences (*p* < 0.01) as determined using Fisher’s protected LSD test.

In addition, CaZingipain2 was found to act positively in pepper immunity against RSI under WM conditions. We assayed the function of CaZingipain2 by studying the effect of *CaZingipain2* silencing on pepper immunity under WM conditions. A qRT−PCR was used to verify that the silencing was successful (Figure 5A). The TRV::*CaZingipain2* plants that exhibited reduced resistance to RSWM at 2 days post−inoculation (dpi) (Figure 5B) also displayed a higher dynamic disease index from 2 to 12 dpi (Figure 5C). According to previous studies, *CaMgst3* and *CaPRP1* act positively and specifically in pepper immunity against *R. solanacearum* infection under WM conditions [33]. To test whether these genes were involved in CaZingipain2−mediated pepper immunity against *R. solanacearum* infection under WM conditions, we tested *CaMgst3* and *CaPRP1* expression in *CaZingipain2*−silenced pepper plants. The results showed that these two genes were reduced significantly by *Cazingipain2* silencing upon *R. solanacearum* infection under WM conditions compared to the wild−type plants (Figure 5D). These results demonstrate that CaZingipain2 acts positively in pepper immunity against *R. solanacearum* under WM conditions.

### 2.5. Overexpression of CaZingipain2 Promoted Nicotiana Benthamiana Immunity against R. solanacearum under RM and WM Conditions

**Figure 6:**
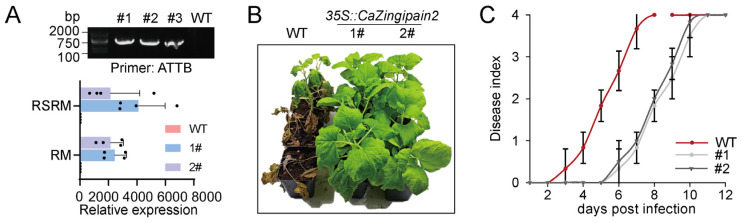
Overexpression of *CaZingipain2* enhanced the resistance of pepper to *R. solanacearum* inoculation at RM. (**A**) Success of overexpressing *CaZingipain2* in transgenic tobacco plants (#1 and #2) by PCR and qRT−PCR. Transcriptional level of each tested gene was normalized to that of *NbActin*. Dots represent four biological replicates (*n* = 4). (**B**) Resistance to RSRM was greater in *CaZingipain2*−overexpressing tobacco plants than in wild−type plants. (**C**) *CaZingipain2*−overexpressing tobacco plants exhibited lower dynamic disease index from 3 to 12 dpi at RM. Ten pepper plants were used for analysis of disease indices over time (*n* = 10).

To further confirm the role of *CaZingipain2* in plant defense against RSI, we created *CaZingipain2*−overexpressing T_2_ *N. benthamiana* lines, among which two lines were randomly selected for further use. *CaZingipain2* expression in the two lines was confirmed by PCR and qRT−PCR (Figure 6A). Compared with the control plants, the transgenic tobacco plants with Cazingipain2 showed resistance (Figure 6B) and a lower disease index (Figure 6C) when inoculated with *R. solanacearum* under RM conditions. These results suggest that *CaZingipain2* overexpression enhanced the resistance of tobacco plants to RSRM.

**Figure 7:**
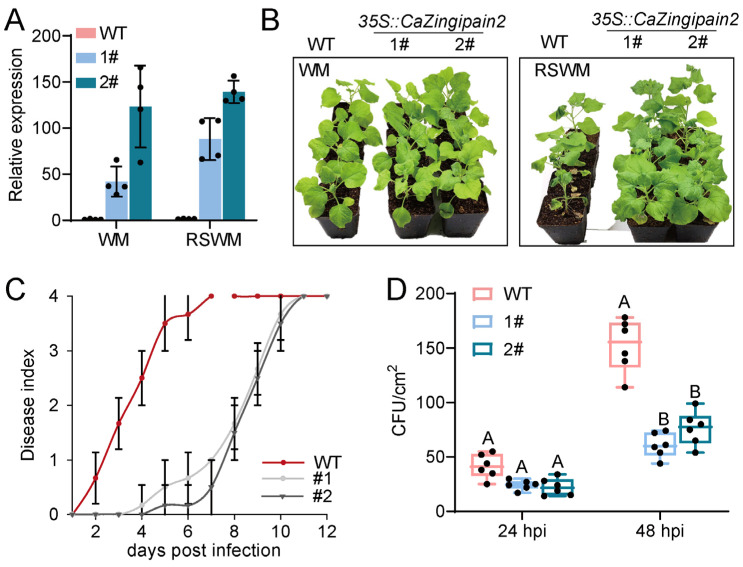
The overexpression of *CaZingipain2* enhanced the resistance of pepper to *R. solanacearum* inoculation at WM. (**A**) The success of *CaZingipain2* overexpression in transgenic tobacco plants (#1 and #2) by qRT−PCR. The transcriptional level of each tested gene was normalized to that of *NbActin*. The dots represent four biological replicates (*n* = 4). (**B**) The resistance to RSWM was greater in the *CaZingipain2*−overexpressing tobacco plants than in the wild−type plants, while the *CaZingipain2*−overexpressing and wild−type plants did not exhibit a damaged phenotype upon exposure to WM. (**C**) The *CaZingipain2*−overexpressing tobacco plants exhibited a lower dynamic disease index from 2 to 12 dpi at RM. Ten pepper plants were used for an analysis of disease indices over time (*n* = 10). (**D**) *CaZingipain2* overexpression repressed *R. solanacearum* growth in tobacco plants challenged with *R. solanacearum* at WM. The dots represent six biological replicates (*n* = 6). The different uppercase letters indicate that the means are highly significantly different (*p* < 0.01), as determined using Fisher’s protected LSD test.

We first demonstrated that *CaZingipain2* overexpression in tobacco was successful at high temperatures by qRT−PCR (Figure 7A). Upon *R. solanacearum* inoculation under WM conditions, the transgenic *N. benthamiana* plants exhibited higher levels of disease resistance (Figure 7B), as reflected by a lower disease index (Figure 7C) and lower bacterial growth (Figure 7D). These results suggest that *CaZingipain2* expression enhanced the resistance of tobacco plants to *R. solanacearum* infection under WM conditions. This result further confirmed the results from the gene silencing in pepper plants that *CaZingipain2* acts positively in pepper immunity against *R. solanacearum* under WM conditions.

## 3. Discussion

Although immunity against *R. solanacearum* infection in pepper and tomato plants is temperature−dependently regulated, an understanding of immunity in these plants under warm−temperature and moist conditions, and the association with immunity at room temperature remains elusive. The data in this study indicate that CaZingipain2 is upregulated and acts positively in pepper plants upon *R. solanacearum* infection at room temperature, as well as under WM conditions.

Our data show that the *CaZingipain2* transcript level was much higher in pepper plants upon *R. solanacearum* infection under WM and RM conditions than in the mock treatment, implying its involvement in pepper immunity against *R. solanacearum* infection under WM conditions, as the genes upregulated in pepper plants upon *R. solanacearum* infection have generally been found to be involved in pepper immunity against *R. solanacearum* [34,35,36,37,38,39]. This speculation was further confirmed by the gene silencing experiment, in which *CaZingipain2* silencing significantly reduced the susceptibility of pepper plants to *R. solanacearum* infection not only under RM conditions, but also under WM conditions. By contrast, *CaZingipain2* overexpression significantly reduced the susceptibility of *Nicotiana benthamiana* plants to *R. solanacearum* infection not only under WM conditions, but also under RM conditions. These data collectively indicate that *CaZingipain2* acts positively in pepper immunity against *R. solanacearum* infection under both WM and RM conditions. Our results are consistent with the role of cysteine proteases in plant immunity as a central hub [7,8,9,10], at least partially by releasing damage−associated molecular patterns (DAMPs) or pathogen−associated molecular patterns (PAMPs) that are recognized by receptors, activating immune signaling cascades and consequently immune responses [9]. Many studies have shown that proteases act in the plant immune response mainly by secreting specific proteins to the cells and targeting pathogenic microorganisms [40,41]. However, some cysteine proteases are involved in the recognition of effector proteins by membrane surface receptors, such as RCR3 [42,43]. Unlike them, CaZingipain2 is distributed in the nucleus, suggesting that it may target nuclear proteins for disease resistance. At present, intracellular proteolysis is thought to participate in plants’ defense responses to pathogens, but what protein is targeted by CaZingipain2 still needs to be studied [15].

Bacterial wilt under WM conditions in pepper and other solanaceous plants, including tomato and tobacco, generally tends to be more serious than that at room temperature or under RM conditions [33]. These plants might employ cytokine−mediated immunity to cope with bacterial wilt under WM conditions, while activating SA− and JA−mediated immunity to protect themselves against the disease under RM conditions [15,33,34]. Our data further show that the reduced susceptibility of *CaZingipain2*−silenced pepper plants to *R. solanacearum* under WM conditions is closely related to the downregulation of *CaMgst3* and *CaPRP1*, which act positively and specifically in pepper immunity against *R. solanacearum* infection under WM conditions. Meanwhile, the reduced susceptibility of *CaZingipain2*−silenced pepper plants to *R. solanacearum* under RM conditions is closely related to the downregulation of SA−responsive *CaNPR1* and JA−responsive *CaDEF1*, which act positively and specifically in pepper immunity against *R. solanacearum* infection under RM conditions [33]. These data indicate that *CaZingipain2* acts positively in pepper immunity against *R. solanacearum* at different temperatures by activating different immune signaling pathways. Recent studies have also reported that some genes have bacterial wilt resistance functions at different temperatures, indicating that this phenomenon may be a widespread mechanism of plant resistance to *R. solanacearum* invasion [44]. This differential activation of pathways at different temperatures might be attributable to some unidentified regulatory proteins that are context−specifically activated. Notably, plants use the same protein to activate two different types of immune responses, which undoubtedly benefits the rapid switching from one immune response to another when the temperature changes rapidly.

In conclusion, we identified the role of ginger protease CaZingipain2 in the defense against *R. solanacearum* infection in pepper plants at different temperatures, which lays a foundation for further research on the resistance mechanism of pepper plants to bacterial wilt.

## 4. Materials and Methods

### 4.1. Plant Materials and Growth Conditions

Seeds of the inbred pepper lines HN42 and TT5203, as well as of tobacco, were sown in a soil mix [peat moss/roseite, 3:1 (*v*/*v*)] (PINDSTRUP, Denmark) in plastic pots (7 cm × 7 cm × 7.3 cm), and placed in a growth room at 28 °C, 60–70 mmol photons m^−2^ s^−1^, a relative humidity of 70%, and a 16 h light/8 h dark cycle.

### 4.2. Sequence Analysis and Primer Design

The ORF sequence of an objective gene was obtained from the NCBI, and ORF and virus−induced gene silencing (VIGS) primers were designed with DNAMAN 8 software based on Gateway technology. Fluorescent quantitative primers were designed according to the online software https://www.primer3plus.com/ (accessed on 10 January 2018).

### 4.3. Vector Construction

To construct a vector for the overexpression of an objective gene, its full−length ORF was amplified by PCR using specific primer pairs (Table S1). The CDS fragment was cloned and inserted into the entry vector pDONR207 by a BP reaction using a Gateway system (Invitrogen, 11789020). To construct the vectors for overexpression and subcellular location assays, the ORFs were further linked to the pEarleyGate plasmid vectors pEarlyGate101 and pEarlyGate103 [45] via LR reactions. In addition, to construct a vector for silencing a given gene, a specific fragment of its CDS was amplified by PCR using specific primer pairs (Table S1), and inserted into the destination vector pPYL279 via an LR reaction.

### 4.4. Virus−Induced Gene Silencing (VIGS) Assay

**Figure 8:**
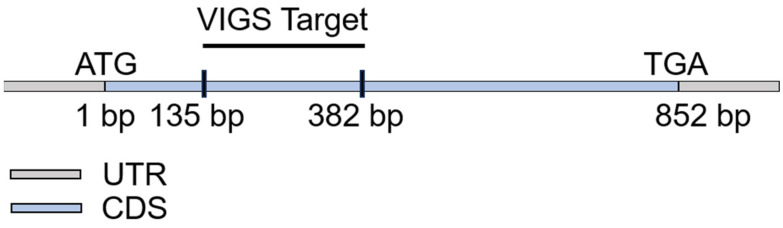
Diagram of the fragments in the mRNA of *CaZingipain* used for VIGS.

To test the function of the objective gene in the knockdown mutants, a VIGS was performed following a previously described method [39]. A VIGS primer for *CaZingipain2* was designed through the Solanidae database Sol Genomics Network, with a range of about 150–380 bp (Figure 8). Either pTRV2::*00*, pTRV2::*CaPDS*, or pTRV2::OG (objective gene) was transformed into Agrobacterium tumefaciens strain GV3101 cells by cold melting [46]. Then, it was mixed with pTRV1 at a 1:1 ratio and then incubated at 28 °C at 60 rpm for 2 h. The mixed solution was injected into the cotyledons of 2−week−old pepper plants, which were then placed in the dark for 56 h at 16 °C.

### 4.5. Subcellular Localization

Tobacco leaves were infected with *35S::CaZingipain*–*GFP* by an Agrobacterium−mediated transformation. After 48 h, the infected tobacco leaves were cut to 1 cm^2^ and placed on slides. Laser scanning confocal microscopy was used to observe the position of the fluorescence signals in the cells, and the observation results were photographed and preserved.

### 4.6. Genetic Transformation of Nicotiana Benthamiana

The genetic transformation of tobacco was performed using the methods of Regner et al. [47] and Bardonn et al. [48]. Leaf disks were transformed with GV3101 cells containing the target vector, and T_0_ plants obtained by screening with 10% PPT (glyphosate, Sigma, 45520) were confirmed by PCR with specific primers (Table S1). The confirmed T_0_ plants were self−pollinated to produce T_1_ line seeds, which were harvested separately, and the obtained seeds were selected with 10% PPT at germination. Similarly, the seeds of the T_2_ and T_3_ lines were obtained, and the genes tested were analyzed functionally using the purebred T_3_ line plants.

### 4.7. Total RNA Extraction and Reverse Transcription

As described in our previous study [15], the total RNA was isolated using 1.5 mL RNase−free microcentrifuge tubes, four stainless steel beads, and Tissue Lyser II (Qiagen, Dusseldorf, Germany) to destroy the frozen plant material in liquid nitrogen. The total RNA was isolated with TRIzol (Invitrogen, Carlsbad, USA) and chloroform, and the RNA was precipitated with isopropanol. The RNA was washed with 75% ethanol. Finally, it was centrifuged at 7500 r/m for 5 min at 4 °C, the supernatant was discarded, dried in an ultra−clean medium, dissolved in RNase−free water, and the quality of the RNA solution obtained was tested.

The RNA concentration and mass were determined using a NanoDrop 2000 spectrophotometer (Thermo Scientific, Waltham, MA, USA). A reverse transcription kit was supplied by Promega. Briefly, 500 ng of total RNA was reverse−transcribed into single−stranded cDNA. After the reverse transcription was completed, the obtained product was diluted ten times and used for subsequent fluorescence quantitative PCR analysis experiments.

### 4.8. Fluorescent Quantitative PCR

A SYBR^®^ Premix Ex TaqTM II kit provided by Takara was used to quantitatively detect the expression of the target gene at the transcription level by the SYBR Green I method. The target cDNA obtained by reverse transcription was used as the template. See Table S1 for the relevant primers. A reaction system of 10 μL was used, and included cDNA, 1 μL; primer, 0.2 μL; ddH_2_O, 3.6 μL; and 2×SYBR, 5 μL. The reaction program settings were 94 °C for a 5 s pre−denaturation; 94 °C for 30 s, 60 °C for 34 s, and cycled 40 times. Four biological replicates were performed for each treatment.

### 4.9. Colony−Forming Unit (CFU) Determination

In order to test the growth of *R. solanacearum* in the plants, bacterial colony units were measured in the plant material inoculated with *R. solanacearum* for 4 biological replicates per group. At 48 h and 96 h, plant samples were collected around the inoculation site, flushed into 1.5 mL centrifuge tubes, and 1mL ddH_2_O was added. The sample was further ground with a small stick, and 100 μL of pathogenic bacteria suspension was diluted 10^−3^, 10^−4^, and 10^−5^ in turn. A 200 μL amount of bacterial solution was then added to solid TTC medium and incubated in an incubator at 28 °C for 48 h.

### 4.10. Disease Index

For the disease index determination, the root−irrigation inoculation of *R. solanacearum* was performed by adding 10 mL of the bacterium solution (OD_600_ = 0.5) into each pot with one pepper plant or *Nicotiana benthamiana* plant. The pots were then placed in the illumination incubator at 28 °C and 80% relative humidity. After treatment, the phenotype was observed and the disease index was calculated according to Table 1.

After *R. solanacearum* inoculation, the disease level of the plants was recorded, and the disease index was output through GraphPad Prism8.

## Figures and Tables

**Table 1:** Grading standards for evaluation of disease resistance of pepper plants to R. solanacearum by root irrigation.

Disease Level	Symptom Description
0	Plant normal, no symptoms.
1	Plant is slightly wilted, 1/4 of the leaves are wilted.
2	One−half of the leaves are wilted.
3	Three−fourths of the leaves are wilted.
4	Whole plant wilts, or plant dies.

## Data Availability

The data that support the findings of this study are available in the Supporting Information.

## Supplementary Materials

The following supporting information can be downloaded at https://www.mdpi.com/article/10.3390/plants13182552/s1, Table S1: Oligonucleotides used in this study.
